# Adjuncts or adversaries to shared decision-making? Applying the Integrative Model of behavior to the role and design of decision support interventions in healthcare interactions

**DOI:** 10.1186/1748-5908-4-73

**Published:** 2009-11-12

**Authors:** Dominick L Frosch, France Légaré, Martin Fishbein, Glyn Elwyn

**Affiliations:** 1 Department of Medicine, Division of General Internal Medicine & Health Services Research, University of California, Los Angeles, USA; 2 Department of Health Services Research, Palo Alto Medical Foundation Research Institute, Palo Alto, CA, USA; 3 Department of Family Medicine, Université Laval, Québec City, Canada; 4 Annenberg Public Policy Center, Annenberg School for Communication, University of Pennsylvania, Philadelphia, USA; 5 Department of Primary Care and Public Health, School of Medicine, Cardiff University, UK

## Abstract

**Background:**

A growing body of literature documents the efficacy of decision support interventions (DESI) in helping patients make informed clinical decisions. DESIs are frequently described as an adjunct to shared decision-making between a patient and healthcare provider, however little is known about the effects of DESIs on patients' interactional behaviors-whether or not they promote the involvement of patients in decisions.

**Discussion:**

Shared decision-making requires not only a cognitive understanding of the medical problem and deliberation about the potential options to address it, but also a number of communicative behaviors that the patient and physician need to engage in to reach the goal of making a shared decision. Theoretical models of behavior can guide both the identification of constructs that will predict the performance or non-performance of specific behaviors relevant to shared decision-making, as well as inform the development of interventions to promote these specific behaviors. We describe how Fishbein's Integrative Model (IM) of behavior can be applied to the development and evaluation of DESIs. There are several ways in which the IM could be used in research on the behavioral effects of DESIs. An investigator could measure the effects of an intervention on the central constructs of the IM - attitudes, normative pressure, self-efficacy, and intentions related to communication behaviors relevant to shared decision-making. However, if one were interested in the determinants of these domains, formative qualitative research would be necessary to elicit the salient beliefs underlying each of the central constructs. Formative research can help identify potential targets for a theory-based intervention to maximize the likelihood that it will influence the behavior of interest or to develop a more fine-grained understanding of intervention effects.

**Summary:**

Behavioral theory can guide the development and evaluation of DESIs to increase the likelihood that these will prepare patients to play a more active role in the decision-making process. Self-reported behavioral measures can reduce the measurement burden for investigators and create a standardized method for examining and reporting the determinants of communication behaviors necessary for shared decision-making.

## Background

Over the last several decades, we have witnessed a redefinition of the role of patients in decision-making. This new conceptualization of the role of patients in decision-making in healthcare settings is often termed 'shared decision-making' [1-3]. Shared decision-making as a goal for clinical consultations has been clearly distinguished from the traditional paternalistic model in which the physician is the primary decision-maker and the patient is expected to follow the directives of the physician. [1,4]. Numerous authors have contributed important conceptual descriptions of shared decision-making which have succeeded in identifying behaviors that physicians and patients need to engage in, in order for shared decision-making to occur [5-12]. The key point for researchers interested in the development of interventions for facilitating shared decision-making is that many of these behaviors may be contextually new for patients. In that sense, a critical component of any decision support intervention (DESI) is the degree to which it not only provides information about the decision in question, but also the degree to which the intervention facilitates adoption of these new behaviors by patients.

Despite the growing interest in shared decision-making, the current clinical reality is that much room for growth remains in shifting encounters between physicians and patients from paternalism to this new model. [13]. Partly in response to this, researchers have devoted significant effort to developing interventions to facilitate shared decision-making. [14]. Although some work has focused on physicians, the majority of interventions are developed for patients, mostly in the form of DESIs. [15,16]. The purpose of a DESI is to assist patients in making a specific and deliberate choice among different options together with their physician to address a clinical problem [15]. A systematic review aggregating the results from 55 randomized trials of DESIs found that compared to usual care or an informational leaflet, exposure to a DESI improved a number of cognitive variables, such as increasing patient knowledge and realistic expectations about what a clinical option could and could not accomplish, and lowering decisional conflict. Individuals who viewed a DESI were less likely to remain undecided and often made different decisions after reviewing a DESI [17]. While these findings are important in building the evidence base supporting DESIs, whether or not they actually prepare patients to engage in the behaviors necessary for shared decision-making with their physicians remains largely unexplored [18,19]. Studies that explore the impact of DESIs on interactional behaviors in subsequent consultations are needed.

### Advancing DESI research

We believe that a substantial barrier to advances in implementing shared decision-making in routine clinical care settings is the lack of theoretical and conceptual clarity about what it is DESIs are trying to accomplish, and whether interventions are designed to facilitate the behaviors necessary for shared decision-making or are based on other assumptions, *e.g*., that giving patients information about their options will be sufficient to facilitate shared decision-making [20]. Indeed, the failure to build on existing theory is often cited as one the major sources for lack of effectiveness of interventions in the healthcare context [21-25]. It has been argued that the use of theory will improve our understanding of the underlying mechanisms by which behavior change occurs. This will in turn ensure that effective interventions can be designed and tested with relevant *a priori *research hypotheses. [26]. Therefore, the purpose of the present paper is to go beyond the cognitive processes (*e.g*., patient knowledge) and outcomes of clinical decision-making that are typically the focus of studies on DESIs, and examine how an existing behavioral theory can contribute to the development of interventions that prepare patients to engage in the behaviors that are necessary for shared decision-making to be possible. To that end, we consider the application of a theory of behavior that we believe can guide the development and evaluation of effective interventions to facilitate these specific behaviors necessary for shared decision-making.

## Discussion-behavioral perspectives in DESI research

### What behaviors are necessary for shared decision-making?

A recent systematic review by Makoul and Clayman (2006) examined the published literature around conceptual definitions of shared decision-making and distilled these works into a proposed model of shared clinical decision-making [27]. In formulating this model, the authors distinguished between general qualities of a physician-patient encounter that could be characterized as shared decision-making and specific observable behaviors that are essential elements for shared decision-making to take place and are therefore potential targets for a DESI. Table 1 lists the identified observable behaviors, divided into those that the physician needs to engage in, those the patient needs to engage in, and those both the physician and patient need to engage in [27]. While not at all authors agree on these elements, there is considerable overlap between different conceptualizations of shared decision-making [27].

**Table 1:** Behaviors necessary for shared decision-making

Who engages in the behavior	Observable behavior
Physician	- Defining/explaining the medical problem* - Presenting options for the medical problem* - Making a recommendation

Physician and Patient	- Clarifying understanding - Discussing risks, benefits and costs of options† - Discussing the ability to make a decision† - Making or deferring a decision

Patient	- Expressing values and preferences related to potential health outcomes and options

Adopted from Makoul and Clayman, 2006.

*Primary targets of DESIs to date.

† These are arguably behavioral categories, which include several specific behaviors such as asking questions, expressing opinions, or voicing concern.

To date, DESIs have arguably focused on giving patients information to help them understand the medical problem they are facing, describe the options available to them including their pros and cons, and potentially facilitate patients thinking about how they might weigh the trade-offs between different options relative to their values for different health outcomes. However, shared decision-making requires not only a cognitive understanding of the medical problem and deliberation about the potential options to address it, but also a number of communicative behaviors that the patient and physician need to engage in to reach the goal of making a shared decision. DESIs could incorporate components that would assist patients in adopting the behaviors necessary for shared decision-making, for example by prompting patients to write down questions or modeling communication behaviors with physicians, especially under difficult circumstances such as when a physician is not attending to the patient's perspective [28].

We hypothesize that the most effective interventions to facilitate shared decision-making will target both patients and physicians. As is clear from Table 1, several of the communication behaviors necessary for shared decision-making are interactional behaviors that require engagement from the patient and physician. Even the best prepared patient may not be able to achieve the goal of sharing a clinical decision if the patient's involvement in the process is not supported by the physician. Communication is a dynamic process that involves a give and take between both parties involved [28]. Methodological advances have been made in analyzing dyadic data to determine the relative influence of two individuals on each other in the clinical decision-making process, and investigators could measure behaviors at both the patient and physician levels [29]. However because DESIs are principally targeted at patients, and DESIs that help behaviorally prepare the patient for shared decision-making are more likely to be successful than those that don't, the remainder of our paper focuses on the patient side of the clinical dyad.

### How behavioral theory can guide the development of interventions to increase shared decision-making

The field of psychology has developed a large body of theoretical and empirical work devoted to conceptualizing and testing the determinants of behavior and behavior change. [30]. However, little behavioral research has focused on shared decision-making and what this means from a behavioral perspective at the patient level. Theoretical models of behavior can guide both the identification of constructs that will predict the performance or non-performance of specific behaviors relevant to shared decision-making, as well as inform the development of interventions to promote these specific behaviors.

One challenge for shared decision-making researchers is which among the many different theories to draw on in developing interventions. We focus on the Integrative Model (IM) for several reasons. First, this model of behavior combines the primary constructs of four theories of behavior that have been applied in many health contexts over the past 30 years. [21]. These include the Theory of Reasoned Action, the Theory of Planned Behavior, the Health Belief Model and Social Cognitive Theory. [31-34]. Implicit in the combination of these theories into one IM is that these theories have sometimes used different terminologies for very similar constructs. The strength of these constructs in predicting behavior in a broad variety of contexts is documented by meta-analytic studies and systematic reviews. There are substantial significant relationships between attitudes and behavior [35], self-efficacy and behavior [24], perceived social norms and behavior [36,37], and behavioral intention and behavior [38,39]. Finally, a pragmatic reason for focusing on this model is that it has a well-developed approach for measuring its central constructs of attitudes, perceived normative pressure, and self-efficacy, that can be adapted to an investigator's specific behavior of interest [31,40].

**Figure 1:**
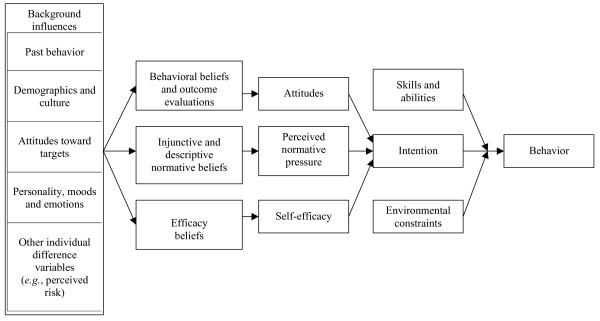
**The Integrative Model (adapted from Fishbein, 2000)**.

Figure 1 provides a graphical overview of the IM. From the perspective of the IM, a behavior is likely to occur if a person has formed an intention to perform that behavior, the person has the skills necessary to perform the behavior, and there are no environmental constraints that prevent the person from performing the behavior. [21]. If a person has not formed an intention to perform a specific behavior, the IM suggests there are three primary determinants of intention. The first is a person's attitude toward performing the behavior; that is, a positive or negative evaluation of personally performing the behavior in question. For example, in making a choice about colon cancer screening, does a patient believe that asking questions about the screening options is wise or foolish; pleasant or unpleasant? Second is a person's perceived normative pressure with respect to performing the behavior. In other words, does a patient perceive that other persons important to them think s/he should (or should not) ask questions about colon cancer screening options, and/or do they believe that others like them are or are not asking questions? Finally, self-efficacy reflects whether a person perceives that they have the necessary skills and abilities to perform the behavior if they really want to do so. Each of these primary constructs is in turn the function of underlying salient beliefs. Attitudes reflect underlying outcome expectancies about whether engaging in a particular behavior will produce favorable or unfavorable outcomes. Perceived normative pressure reflects normative beliefs about what significant others expect the individual to do, as well as beliefs about what these significant others are themselves doing. Self efficacy reflects the salient beliefs that one can perform the behavior given the presence (or absence) of specific barriers or facilitators. For example, can the patient ask questions about colon cancer screening options, even in difficult circumstances such as when the physician is under time pressure. [21]?

The ability of these constructs to predict behavior depends upon how well the behavior is defined and upon the degree of correspondence between the measure of behavior and the measures of the constructs. [21]. This requires a clear distinction between goals, behavioral categories, and specific behaviors. For example, sharing a clinical decision or reaching consensus with a physician is not a behavior, but rather is a goal. Preceding this goal is a category of behaviors that we might term 'engaging in shared decision-making' which in turn consists of several specific behaviors. These specific behaviors need to be further defined with regard to the specific action (*e.g*., expressing one's preferences about a set of options), the target of this action (*e.g*., personal physician), the context (*e.g*., during a consultation about treatment), and the time period during which the behavior should occur (*e.g*., when I see him/her today). [21].

## Practical application

### Applying the IM to the development and evaluation of decision support interventions

Numerous measures for different aspects of shared decision-making have been published, and two systematic reviews have examined this literature [41,42]. Many of these measures focus on patient preferences for the decision-making process and cognitive aspects of decision-making, such as decisional conflict. Existing behavioral measures, including objective measures that require resource intensive audiotaping of clinical encounters, are focused on physician behaviors related to facilitating shared decision-making. Measures of patient behaviors for shared decision-making are lacking [42].

There are several ways in which the IM could be used in research on the behavioral effects of patient interventions to facilitate shared decision-making. An investigator could relatively easily draw on the model to measure the effects of an intervention designed to facilitate shared decision-making on attitudes, normative pressure, self-efficacy, and intentions related to relevant behaviors. As a first step, this would require identifying the behaviors of interest and adapting the IM measures accordingly, accounting for action, target, context, and time period. [21]. Imagine, for example, that an investigator has developed a DESI to help patients decide whether or not they wish to receive colon cancer screening. Beyond informing patients about the options, the intervention is also intended to increase the specific patient behavior of telling their physician their preferences for colon cancer screening options. In this case, the specific action could be defined as 'telling your physician your preferences for colon cancer screening', the target would be the patient's physician, and the time period and context might be 'when you see your physician for a consultation today'. Before we continue here, it is important to note that expressing one's preferences about a set of options is but one of several communicative behaviors required for shared decision-making. Other communication behaviors of interest might include asking questions about the medical problem or treatment options, requesting a recommendation from the physician, or disagreeing with a recommendation given by a physician. Investigators can tailor their measurements according to their substantive behavioral interests.

Attitudes would be measured with semantic differential items that ask the respondents to rate whether telling their physician their preferences would be 'good' or 'bad', 'wise' or 'foolish', 'necessary' or 'unnecessary', 'beneficial' or 'harmful', and 'pleasant' or 'unpleasant'. Perceived normative pressure would be measured with items that assess both the descriptive norms (*i.e*., what the person perceives others as doing) and injunctive norms (*i.e*., what the person perceives others whose opinions are important expect him or her to do). Self-efficacy would be measured with items that ask the respondent to appraise their ability to tell their physician their preferences. Finally, behavioral intention would be measured with items that directly probe the person's intention according to the target, context, and time period of interest [31,40]. Table 2 illustrates the specific questionnaire items an investigator might use to assess the IM measures in relation to telling a physician one's colon cancer screening preferences during a consultation. If an investigator were interested in other communicative behaviors, these could be substituted for expressing preferences about colon cancer screening. An important point to keep in mind is that the psychometric properties of the instrument that is developed need to be assessed to ensure adequate reliability [43]. By using measures such as the ones described in Table 2, the investigator would be able to assess whether the DESI increases the patient's intention to tell their physician their preferences and, if so, what was the specific mechanism of this effect. One important caveat is that even if the effect of the intervention is similar in different patient populations, its mechanism may well vary. [21]. In some populations, the effect may be attitudinally driven, whereas in others it may be normatively driven. However, by only measuring the central constructs of the IM one does not gain any insight into the determinants of these constructs or how the intervention affects these determinants. [21]. If one were to find that the intervention does not produce the desired or expected behaviors, then one would need to delve deeper to understand the determinants of attitudes, perceived normative pressure, and self-efficacy. One could then develop interventions that target these determinants, thereby increasing the likelihood that the intervention would lead to the adoption of the target behavior.

**Table 2:** Sample items for measuring the central direct constructs of the Integrative Model

Construct	Survey items								
Behavioral intention	I intend to tell my doctor about my preferences for colon cancer screening when I see him/her for a consultation today:								
	Strongly disagree	-3	-2	-1	0	1	2	3	Strongly agree
	I am willing to tell my doctor about my preferences for colon cancer screening when I see him/her for a consultation today:								
	Strongly disagree	-3	-2	-1	0	1	2	3	Strongly agree
	I will tell my doctor about my preferences for colon cancer screening when I see him/her for a consultation today:								
	Very unlikely	-3	-2	-1	0	1	2	3	Very likely

Attitudes	My telling my doctor about my preferences for colon cancer screening when I see him/her for a consultation today would be:								
	Harmful	-3	-2	-1	0	1	2	3	Beneficial
	Bad	-3	-2	-1	0	1	2	3	Good
	Unpleasant	-3	-2	-1	0	1	2	3	Pleasant
	Foolish	-3	-2	-1	0	1	2	3	Wise
	Unenjoyable	-3	-2	-1	0	1	2	3	Enjoyable
	Not useful	-3	-2	-1	0	1	2	3	Useful

Perceived normative pressure	Most people who are important to me think that I should tell my doctor about my preferences for colon cancer screening when I see him/her for a consultation today:								
	Strongly disagree	-3	-2	-1	0	1	2	3	Strongly agree
	Most of the people who are important to me would recommend that I tell my doctor about my preferences for colon cancer screening when I see him/her for a consultation today:								
	Strongly disagree	-3	-2	-1	0	1	2	3	Strongly agree
	Most people like me tell their doctors about their preferences for colon cancer screening when they see him/her for a consultation:								
	Strongly disagree	-3	-2	-1	0	1	2	3	Strongly agree
	Other people I know would tell their doctor about their preferences for colon cancer screening when they see him/her for a consultation:								
	Strongly disagree	-3	-2	-1	0	1	2	3	Strongly agree

Self-efficacy	My telling my doctor about my preferences for colon cancer screening when I see him/her for a consultation today would be:								
	Not up to me	-3	-2	-1	0	1	2	3	Up to me
	If I really wanted to, I could tell my doctor about my preferences for colon cancer screening when I see him/her for a consultation today:								
	Strongly disagree	-3	-2	-1	0	1	2	3	Strongly agree
	During my consultation with my doctor today, I will be in control of telling him/her about my preferences for colon cancer screening:								
	Strongly disagree	-3	-2	-1	0	1	2	3	Strongly agree

As noted above, each of the IM constructs is a function of underlying salient beliefs that a person holds about engaging in the behavior of interest. [21]. Identifying these initially requires formative qualitative research with the specific population of interest [31]. The goal of conducting formative research is to elicit salient beliefs underlying each of the central constructs (*i.e*., attitudes, perceived normative pressure, and self-efficacy). Table 3 illustrates the formative research questions an investigator might use to elicit salient beliefs related to expressing preferences about colon cancer screening. [40]. The results shown in Table 3 are hypothetical. For each set of beliefs, the qualitative results are then translated into survey items that can be used to examine the beliefs quantitatively, providing an indirect measure of the central constructs of the model. Attitudes can be assessed indirectly by first calculating the product of each behavioral belief and its related outcome evaluation, and then summing the products into a single score. Perceived normative pressure can be assessed indirectly by calculating the sum of the descriptive and injunctive normative beliefs. Finally, self-efficacy can be assessed indirectly by summing the scores for each efficacy belief into a single score. [40].

**Table 3:** Measuring the salient beliefs underlying attitudes, perceived normative pressure and self-efficacy

Attitudes	Hypothetical results
Formative research questions: What do you believe are the advantages (disadvantages) of telling your doctor today about your preferences for different colon cancer screening options? Is there anything else you associate with telling (not telling) your doctor today about your preferences for different colon cancer screening options?	Advantages: My doctor will know what is important to me Disadvantages: My doctor may think that I lack confidence in his judgment

Survey items assessing behavioral beliefs: If I tell my doctor about my preferences for colon cancer screening when I see him/her for a consultation today, he/she will know what is important to me Unlikely -3 -2 -1 0 1 2 3 Likely If I tell my doctor about my preferences for colon cancer screening when I see him/her for a consultation today, he/she may think that I lack confidence in his/her judgment Unlikely -3 -2 -1 0 1 2 3 Likely	Survey items assessing outcome evaluations: My doctor knowing what is important for me is: Very undesirable -3 -2 -1 0 1 2 3 Very desirable My doctor thinking that I lack confidence in his/her judgment is: Very undesirable -3 -2 -1 0 1 2 3 Very desirable

**Perceived normative pressure**	**Hypothetical results**

Formative research questions: Please list any individuals or groups who would approve (disapprove) of your telling your doctor about your preferences for colon cancer screening. Please list any individuals or groups who tell (do not tell) their doctor about their preferences for colon cancer screening. Are there any other people or groups you associate with telling (not telling) your doctor your preferences about colon cancer screening?	Individuals who approve: Doctor Individuals who disapprove: Wife [because the doctor knows what is best] Individuals who perform the behavior: Colleagues Individuals who do not perform the behavior: Other people my age

Survey items assessing injunctive normative beliefs: My doctor thinks I should not -3 -2 -1 0 1 2 3 should tell him/her about my preferences for colon cancer screening when I see him/her for a consultation today My wife thinks I should not -3 -2 -1 0 1 2 3 should tell my doctor about my preferences for colon cancer screening when I see him/her for a consultation today	Survey items assessing descriptive normative beliefs: Most of my colleagues would tell their doctors their preferences for colon cancer screening when they see him/her for a consultation Unlikely -3 -2 -1 0 1 2 3 Likely Other people my age have told their doctors their preferences for colon cancer screening when they saw him/her for a consultation Unlikely -3 -2 -1 0 1 2 3 Likely

**Self-efficacy**	**Hypothetical results**

Formative research questions: What factors or circumstances would make it easy (difficult or impossible) for you to tell your doctor your preferences for colon cancer screening when you see him/her for a consultation today?	Enabling factors: My doctor asks me what my preferences are Factors that make it difficult or impossible: Not having enough time to talk to my doctor

Survey items assessing the strength of efficacy beliefs: I could tell my doctor my preferences for colon cancer screening when I see him/her for a consultation today even if he/she didn't ask about my preferences Unlikely -3 -2 -1 0 1 2 3 Likely I could tell my doctor my preferences for colon cancer screening when I see him/her for a consultation today even if I have very little time to talk to my doctor. Unlikely -3 -2 -1 0 1 2 3 Likely	

The formative steps described above could be used in two different ways. If an investigator is interested in developing an intervention, these steps can help identify potential targets for a theory-based intervention to maximize the likelihood that it will influence the behavior of interest. Alternately, if an intervention has already been developed and is atheoretical, survey items measuring behavioral beliefs and the related outcome evaluations, injunctive and descriptive normative beliefs, and strength of efficacy beliefs can be used to develop a more fine-grained understanding of the effects of this atheoretical intervention.

### An illustration of the benefit of applying the IM to DESI research

In a recent study, we applied the IM to understanding the effects of a DESI on subsequent patient discussions with their physicians about prostate or colon cancer screening. Patients either reviewed a brief brochure or watched a 30-minute video program about prostate or colon cancer screening in the medical practice immediately prior to a consultation with a physician. Because the video programs are purported to aid the patient in engaging in shared decision-making with their physician, we hypothesized that patients who viewed a video program would be more likely to work with their physicians to make a decision about cancer screening. [44].

Patients completed a questionnaire assessing attitudes, perceived normative pressure, self-efficacy, and behavioral intentions related to 'working with the physician to make a cancer screening decision' after reviewing the DESI, but before seeing the physician. We chose to frame our questions around the behavioral category of 'working with the physician' due to concerns about respondent burden. Before answering the questions, participants read a brief definition of this behavioral category, which was intended to reflect the behaviors that are considered normative of the patient's role in shared decision-making. [44].

Contrary to our hypothesis, we found that a significant number of patients in both groups, who opted against prostate or colon cancer screening, reported not discussing their decisions with their physicians. Although, the differences between the brochure and video groups were not statistically significant, the observed effects were more pronounced among patients who viewed a video. Had we limited our measures to asking patients whether they discussed cancer screening with their physician, we would not have been able to make sense of these unexpected findings. However, by including the IM questions related to working with the physician to make a decision, we were able to identify that patients who watched a video had significantly lower perceived normative pressure and lower intentions to work with their physician to make a decision than patients who reviewed a brochure. Perceived normative pressure about working with the physician was lowest in the group who reviewed a video about prostate cancer screening. Contrary to the brochure, which explicitly encouraged patients to talk to their physician about screening, the video told the patient that 'the decision really depends on what the test means to you' and closed by stating that 'you have to decide if screening is important to you'. Neither the physician testimonials, nor other parts of the video program, explicitly suggested that the decision should be made in direct consultation with the physician. [44].

Thus, with the benefit of theory-enhanced hindsight we learned that our findings weren't necessarily surprising. The DESIs in this study, and the video programs in particular, were not designed to achieve the intervention effects that were intended by the developers. Rather, the video programs were encouraging patients to make the decision for themselves, instead of making a shared decision with their physician. Had the developers of the video DESI explicitly considered the behavioral targets of the intervention and developed it in a theory-driven manner, the program would have arguably included different intervention components that specifically encouraged these behaviors.

### The challenge of behavioral and contextual specificity

The utility and validity of applying behavioral theory to intervention research is related directly to the specificity of the target behavior. The challenge here is that, as noted before, shared decision-making requires several different behaviors on the part of patients and physicians. Correspondingly, investigating the effect of an intervention on each of the relevant behaviors will substantially increase the respondent burden, as the length of a survey to assess the constructs of the IM and its related determinants will be multiplied by the number of behaviors an investigator is interested in. [40]. There are two potential solutions to this problem. On the one hand, an investigator could focus the survey on the behavior that is most difficult for individuals to engage in. The assumption is that if an intervention can affect the behavior that is most difficult, it is also likely to have an effect on other related specific behaviors, although this needs to be tested empirically. An alternative compromise (as described above) would be for an investigator to design survey items around a behavioral category and provide respondents with a clear definition of what specific behaviors are included and targeted by the behavioral category. This will reduce respondent burden, however, the resulting data will lose precision and specificity, which may pose challenges if an intervention does not work as intended [45].

The second challenge for investigators grows from the contextual specificity required by the theory. This relates both to the context for the behavior of interest as well as to the population that the investigator is interested in. For example, a patient may perceive engaging in shared decision-making behaviors with a trusted primary care physician very differently than with a specialist who is providing consultation for the management of a particular medical problem. Similarly, a patient may feel very differently about engaging in these behaviors depending on whether the medical issue being considered is a preventive service, management of a chronic condition, or treatment of an acute condition. Finally, different populations of patients may vary with regard to beliefs underlying their behaviors and the interrelationships between the central constructs of the theory. [21].

## Conclusion

More than a decade of research on DESIs has clearly demonstrated that they have significant positive impacts on the cognitive dimensions of patient involvement in clinical decision-making [17]. Although important questions remain in the cognitive realm [46], it is also important that investigators begin to examine how DESIs can impact interactional behavior, both on the part of patients and physicians. At this time, we simply do not know what behavioral effects DESIs used by a patient before a consultation have in the subsequent clinical encounter. We may find that DESIs do indeed facilitate shared decision-making. Alternately, we may find that DESIs do not fulfill this goal, which will return us to the question of the purpose of DESIs-adjunct to facilitate shared decision-making or adversary that enables patients to make decisions on their own? More research is needed to begin answering this question.

In this paper we have attempted to elucidate how shared decision-making researchers could make use of a widely used behavioral theory that has strong empirical support from the patients' perspective. Our review of the specific ways in which the theory can be applied to research on interventions to facilitate shared decision-making has been necessarily brief. Investigators who are interested in applying the IM can consult other published materials referenced in this article for more detailed guidance on each of the steps involved [21,31,40]. A major obstacle to studying shared decision-making behaviors is that these occur during a consultation between a physician and patient that is challenging to observe directly. Audio- or videotaping patient-physician encounters is a potential solution to this problem, however, this can produce the Hawthorne effect, whereby behavior changes because the individuals know they are being observed. [47]. The IM provides an alternative way of addressing the challenge of the observability of the behaviors because the relationship between behavioral intention and actual behavior, while not perfect, has been shown to be significant [38,39]. Finally, this would create a standardized method for reporting the determinants of target behaviors, and would thus improve our collective knowledge base in this regard.

## Summary

A growing literature documents the efficacy of DESIs in helping patients make informed decisions about healthcare services. DESIs are said to prepare patients for engaging in shared decision-making with their healthcare providers, but little is known about the impact of DESIs on patient communication behavior during a medical consultation. Behavioral theory can guide the development and evaluation of DESIs to increase the likelihood that these will prepare patients to play a more active role in the decision-making process. The use of theory-based behavioral measures in a recent study of DESIs identified a mismatch between the goals and effects of the intervention tested. Self-reported behavioral measures can reduce the measurement burden for investigators and create a standardized method for examining and reporting the determinants of communication behaviors necessary for shared decision-making.

## Competing interests

DLF serves as a consultant for the Foundation for Informed Medical Decision Making, which develops DESIs for patients. The Foundation for Informed Medical Decision Making had no involvement in the writing of this article or the decision to submit it for publication. The authors declare that they have no additional competing interests.

## Authors' contributions

DF, GE and FL conceived the ideas for this article. DF, FL, GE and MF drafted the manuscript. All authors read and approved the final manuscript.

## References

[B1] Frosch DL Kaplan RM Shared decision making in clinical medicine: past research and future directions American Journal of Preventive Medicine 1999 17 285 94 10.1016/S0749-3797(99)00097-5 10606197

[B2] O'Connor AM Llewellyn-Thomas HA Flood AB Modifying unwarranted variations in health care: shared decision making using patient decision aids Health Aff (Millwood) 2004 Suppl Web Exclusive VAR63 VAR72 15471770 10.1377/hlthaff.var.63

[B3] Wennberg JE Fisher ES Skinner JS Geography and the debate over Medicare reform Health Aff (Millwood) 2002 Suppl Web Exclusives W96 114 12703563 10.1377/hlthaff.w2.96

[B4] Kaplan RM Frosch DL Decision making in medicine and health care Annual Reviews in Clinical Psychology 2005 1 525 556 10.1146/annurev.clinpsy.1.102803.144118 17716098

[B5] Charles C Gafni A Whelan T Shared decision-making in the medical encounter: what does it mean? (or it takes at least two to tango) Soc Sci Med 1997 44 681 692 10.1016/S0277-9536(96)00221-3 9032835

[B6] Charles C Whelan T Gafni A What do we mean by partnership in making decisions about treatment? BMJ 1999 319 780 782 10488014 10.1136/bmj.319.7212.780 PMC1116606

[B7] Charles C Gafni A Whelan T Decision-making in the physician-patient encounter: revisiting the shared treatment decision-making model Soc Sci Med 1999 49 651 661 10.1016/S0277-9536(99)00145-8 10452420

[B8] Coulter A Partnerships with patients: the pros and cons of shared clinical decision-making J Health Serv Res Policy 1997 2 112 121 10180362 10.1177/135581969700200209

[B9] Coulter A Entwistle V Gilbert D Sharing decisions with patients: is the information good enough? BMJ 1999 318 318 322 9924064 10.1136/bmj.318.7179.318 PMC1114785

[B10] Coulter A Patient information and shared decision-making in cancer care Br J Cancer 2003 89 Suppl 1 S15 S16 10.1038/sj.bjc.6601080 12915899 PMC2753004

[B11] Elwyn G Edwards A Gwyn R Grol R Towards a feasible model for shared decision making: focus group study with general practice registrars BMJ 1999 319 753 756 10488002 10.1136/bmj.319.7212.753 PMC28229

[B12] Elwyn G Edwards A Kinnersley P Grol R Shared decision making and the concept of equipoise: the competences of involving patients in healthcare choices Br J Gen Pract 2000 50 892 899 11141876 PMC1313854

[B13] Braddock CH3 Edwards KA Hasenberg NM Laidley TL Levinson W Informed decision making in outpatient practice: time to get back to basics Jama 1999 282 2313 20 10.1001/jama.282.24.2313 10612318

[B14] Barry MJ Health decision aids to facilitate shared decision making in office practice Annals of Internal Medicine 2002 136 127 35 11790064 10.7326/0003-4819-136-2-200201150-00010

[B15] Elwyn G O'Connor A Stacey D Volk R Edwards A Coulter A Developing a quality criteria framework for patient decision aids: online international Delphi consensus process BMJ 2006 333 417 10.1136/bmj.38926.629329.AE 16908462 PMC1553508

[B16] Towle A Godolphin W Framework for teaching and learning informed shared decision making BMJ 1999 319 766 771 10488010 10.1136/bmj.319.7212.766 PMC1116602

[B17] O'Connor AM Stacey D Entwistle V Llewellyn-Thomas H Rovner D Holmes-Rovner M Decision aids for people facing health treatment or screening decisions Cochrane Database Syst Rev 2003 CD001431 12804407 10.1002/14651858.CD001431

[B18] Guimond P Bunn H O'Connor AM Jacobsen MJ Tait VK Drake ER Validation of a tool to assess health practitioners' decision support and communication skills Patient Educ Couns 2003 50 235 45 10.1016/S0738-3991(03)00043-0 12900093

[B19] Kaner E Heaven B Rapley T Murtagh M Graham R Thomson R Medical communication and technology: a video-based process study of the use of decision aids in primary care consultations BMC Med Inform Decis Mak 2007 7 2 10.1186/1472-6947-7-2 17214891 PMC1781432

[B20] Legare F Elwyn G Fishbein M Fremont P Frosch D Gagnon MP Translating shared decision-making into health care clinical practices: Proof of concepts Implement Sci 2008 3 2 10.1186/1748-5908-3-2 18194521 PMC2265300

[B21] Fishbein M The role of theory in HIV prevention AIDS Care 2000 12 273 278 10.1080/09540120050042918 10928203

[B22] Fishbein M Hennessy M Kamb M Bolan GA Hoxworth T Iatesta M Using intervention theory to model factors influencing behavior change. Project RESPECT Eval Health Prof 2001 24 363 384 10.1177/01632780122034966 11817197

[B23] Fishbein M Ajzen I Theory-based behavior change interventions: comments on Hobbis and Sutton J Health Psychol 2005 10 27 31 10.1177/1359105305048552 15576497

[B24] Godin G Kok G The theory of planned behavior: a review of its applications to health-related behaviors Am J Health Promot 1996 11 87 98 10163601 10.4278/0890-1171-11.2.87

[B25] Rychetnik L Frommer M Hawe P Shiell A Criteria for evaluating evidence on public health interventions J Epidemiol Community Health 2002 56 119 127 10.1136/jech.56.2.119 11812811 PMC1732065

[B26] Hardeman W Johnston M Johnston D Bonetti D Wareham N Kinmonth A Application of the Theory of Planned Behaviour in Behaviour Change Interventions: a Systematic Review Psychology and Health 2002 123 158

[B27] Makoul G Clayman ML An integrative model of shared decision making in medical encounters Patient Educ Couns 2006 60 301 312 10.1016/j.pec.2005.06.010 16051459

[B28] Street RL Jr Aiding medical decision making: a communication perspective Med Decis Making 2007 27 550 553 10.1177/0272989X07307581 17921451

[B29] Leblanc A Kenny DA O'Connor AM Legare F Decisional Conflict in Patients and Their Physicians: A Dyadic Approach to Shared Decision Making Med Decis Making 2009 29 1 61 8 10.1177/0272989X08327067 19196706

[B30] Glanz K Rimer B Marcus Lewis F Health Behavior and Health Education: Theory, Research, and Practice 2002 San Francisco: Jossey-Bass

[B31] Ajzen I Fishbein M Understanding attitudes and predicting social behavior 1980 Englewood Cliffs, NJ: Prentice-Hall, Inc

[B32] Ajzen I Attitudes, personality and behavior 1988 Open University Press

[B33] Bandura A Social foundations of thought and action: a social cognitive theory 1986 Englewood Cliffs, NJ: Prentice-Hall, Inc

[B34] Janz NK Becker MH The Health Belief Model: a decade later Health Educ Q 1984 11 1 47 6392204 10.1177/109019818401100101

[B35] Kim MS Hunter JE Attitude Behavior Relations - A Metaanalysis of Attitudinal Relevance and Topic Journal of Communication 1993 43 101 142 10.1111/j.1460-2466.1993.tb01251.x

[B36] Rivis A Sheeran P Descriptive norms as an additional predictor in the Theory of Planned Behaviour: A meta-analysis Current Psychology 2003 22 218 233 10.1007/s12144-003-1018-2

[B37] Smith-McLallen A Fishbein M Predictors of intentions to perform six cancer-related behaviours: roles for injunctive and descriptive norms Psychol Health Med 2008 13 389 401 10.1080/13548500701842933 18825578

[B38] Kim MS Hunter JE Relationships Among Attitudes, Behavioral Intentions, and Behavior - A Metaanalysis of Past Research .2 Communication Research 1993 20 331 364 10.1177/009365093020003001

[B39] Webb TL Sheeran P Does changing behavioral intentions engender behavior change? A meta-analysis of the experimental evidence Psychol Bull 2006 132 249 268 10.1037/0033-2909.132.2.249 16536643

[B40] Francis JJ Eccles MP Johnston M Walker A Grimshaw J Foy R Constructing questionnaires based on the Theory of Planned Behavior - A manual for health services researchers. 2004 Newcastle upon Tyne, England, Centre for Health Services Research

[B41] Dy SM Instruments for evaluating shared medical decision making: a structured literature review Med Care Res Rev 2007 64 623 649 10.1177/1077558707305941 17804824

[B42] Simon D Loh A Harter M Measuring (shared) decision-making-a review of psychometric instruments Z Arztl Fortbild Qualitatssich 2007 101 259 267 17601182 10.1016/j.zgesun.2007.02.029

[B43] Cortina JM What is Coefficient Alpha? An Examination of Theory and Application Journal of Applied Psychology 1993 78 98 104 10.1037/0021-9010.78.1.98

[B44] Frosch DL Legare F Mangione CM Using decision aids in community-based primary care: a theory-driven evaluation with ethnically diverse patients Patient Educ Couns 2008 73 490 496 10.1016/j.pec.2008.07.040 18771875 PMC2892794

[B45] Legare F Godin G Guilbert E Laperriere L Dodin S Determinants of the intention to adopt hormone replacement therapy among premenopausal women Maturitas 2000 34 211 218 10.1016/S0378-5122(99)00100-0 10717486

[B46] Nelson WL Han PK Fagerlin A Stefanek M Ubel PA Rethinking the objectives of decision aids: a call for conceptual clarity Med Decis Making 2007 27 609 618 10.1177/0272989X07306780 17873251

[B47] Landsberger HA Hawthorne Revisited 1958 Ithaca, NY: Cornell University

